# Inhibition of HDACs Suppresses Cell Proliferation and Cell Migration of Gastric Cancer by Regulating E2F5 Targeting BCL2

**DOI:** 10.3390/life11121425

**Published:** 2021-12-17

**Authors:** Arshad Ali, Ayaz Ali, Shaker Khan, Muhammad Ibrahim, Mohammed Ali Alshehri, Anand Thirupathi

**Affiliations:** 1 Faculty of Sports Science, Ningbo University, Ningbo 315211, China; 2 School of Life Science, Northwestern Polytechnical University, Xi’an 710072, China; 3 Department of Medical Laboratory Science and Biotechnology, China Medical University, Taichung 406040, Taiwan; u106301102@cmu.edu.tw; 4 Department of Cell Biology and Genetics, Xian Jiao Tong University, Xi’an 710061, China; shakerkhan@stu.xjtu.edu.cn; 5 Department of Pediatrics, Lady Reading Hospital, Peshawar 25000, Pakistan; info@lrh.edu.pk; 6 Department of Biology, Faculty of Sciences, University of Tabuk, Tabuk 71491, Saudi Arabia; ma.alshehri@ut.edu.sa

**Keywords:** HDACs, E2F5, migration, proliferation, gastric cancer

## Abstract

(1) Background: Gastric cancer (GC) is the most common high death-rate cancer type worldwide, with an enhanced prevalence and increased rate of mortality. Although significant evidence on surgery strategy has been generated for the treatment of GC, conclusions are still uncertain regarding profound metastatic or persevering gastric cancer. Therefore, it is essential to develop novel and effective biomarkers or therapeutic targets for the diagnosis of GC. Histone deacetylations (HDACs) are important epigenetic regulators that control the aberrant transcription of critical genes that are mainly involved in cell proliferation, cell migration, regulation of the cell cycle, and different signal pathways. (2) Methods: Expression analysis of HDACs family members and E2F5 in gastric cancer cell lines was determined by RT-PCR and Western blotting. The cell proliferation was determined through an MTT assay. Cell migration was determined using a wound-healing assay. Flow cytometry experiments were used to determine cell-cycle analysis. The statistical software OriginPro 2015 (OriginLab, Northampton, MA, USA) was used to analyze data. A *p* value of < 0.05 was regarded as significant. (3) Results: The present study shows that E2F5 expression is upregulated in GC cancer cell lines compared to normal cell lines, and is positively associated with the level of HDACs and BCL2. HDACi and knocking down of E2F5 as tumor suppressors inhibited cell proliferation, migration invasion, and blocked the cell cycle in gastric cancer cells by suppressing BCL2. The results conclude that the anticancer mechanism of HDACi was determined by regulating E2F5 via targeting BCL2. (4) Conclusions: Our results suggest that the HDAC–E2F5–BCL2 signaling axis might be a novel potential biomarker in gastric cancer.

## 1. Introduction

Gastric cancer is the most common destructive tumor in the digestive tract, with increased morbidity and mortality, mostly in developing countries [1]. Therefore, prognostic identification of indicators related to malignant tumors of the stomach is important in order to understand the clinical outcomes and distinctive treatment plans for patients with gastric cancer. Histone deacetylases (HDACs) have been illustrated to play a key role corresponding to proliferation, migration, and invasion in different types of cancer [2]. HDACs are an enzyme family, and their activity rules the lysine residues of proteins in the acetylation state, notably those present in the amino-terminal extensions of the core histones. The expression of a gene is exaggerated by acetylation of histones, as is its impact on chromatin configuration; the most important role of this enzyme is controlling the cell cycle, cell survival, cell progression, and differentiation [3].

For gastric cancer patients, HDAC inhibitors are being developed as drugs and are showing increased efficacy. Any protein that bonds with HDACs, therefore, has the potential to coactivate the function of enzymes. Individual HDACs interfere with the expression or function of cells to obtain biological reactions [4]. Since HDACs play a crucial role in many pathological processes, their retardation could supply clinical benefits. This has been comprehensively demonstrated for cancer, but there is a desire for the adjacent compounds to prove useful in various human diseases [5]. However, understanding the important functions of specific HDACs in cell proliferation and survival is important to understanding their role in the regulation of many other bioprocesses, which requires further intensive investigations. HDACs have extensive protein deacetylase activity, they have deacetylase lysine residues in non-histone proteins such as microtubules, and they are tumor suppressors [6]. Abnormally regulated HDAC activity has been recognized in cancer, and normal tissues manufacturing HDACs are attractive molecular targets in the search for the mechanisms to cure cancer [7]. Various compact molecular HDAC inhibitors are currently in development as probable anticancer therapeutics [1].

Although there have been some in-depth studies, the specific molecular mechanisms of gastric cancer have not been fully evaluated. The inactivation of HDACs in gastric cancer acts as a core transcriptional mechanism that manages the development of the cell cycle and cyclins’ regulation. Interestingly, E2F5 can enhance and inhibit cell proliferation, transcription, activation, or repress the cell transcriptions that depend on the cell environment. In essence, E2F5 connects the cell cycle to the post-transcriptional mechanism and plays a crucial part in controlling cell growth and the transformation of biological processes that are incriminated in cancer development [8,9]. The BCL2 gene encodes a 26-KDa protein established in the nuclear envelope, and is isolated mostly in mitochondrial layers. In addition, BCL2 blocks apoptosis without disturbing cellular migration and proliferation. This protein also enhances cell survival and subsequently suppresses apoptosis. Interestingly, BCL2 plays a vital role in the control of apoptosis due to protection of cell death, influences by several factors such as radiation, chemotherapy, or deprivation of growth factors [10,11]. However, its role in the pathogenesis of cancer is still poorly elucidated. In the corresponding study, we investigated the anticancer mechanism of HDAC inhibitor and the importance of the HDAC–E2F5–BCL2 axis in the control of cell invasion, proliferation, and suppression along with the migration of gastric tumors.

## 2. Materials and Methods

### 2.1. Cell Culture, Treatment, and Transfection

Cell lines, including MGC-803, MKN28, MKN45, KATO III, AGS, N87, and GES-1, were obtained from Bioresource Collection and Research Center (Hsinchu, Taiwan). All cell lines were maintained and subcultured in RPMI 1640 (Gibco^®^, Thermo Fisher, Waltham, MA, USA) and F-12K medium supplemented with 10% fetal calf serum (HyClone, Logan, Australia) in a humidified 37 °C chamber with 5% CO_2_. The cell culture medium was replaced every 4 h during subculture and the culture plate was washed with Dulbecco’s phosphate-buffered saline (PBS; Gibco^®^, Auckland, New Zealand). KATO-III and N87 cells were treated with histone deacetylase inhibitor (HDACi) and transfected with E2F5 and NC siRNA purchased from Ribobio (Guangzhou, China). The compositions of HDACi are detailed in Supplementary Table S1. The sequences of E2F5 and NC siRNAs are provided in Supplementary Table S2.

### 2.2. RNA Extraction, cDNA Synthesis, and qRT-PCR

Total cellular RNA from the abovementioned cell lines was extracted and purified using a Total RNA extraction kit 1 (TAKARA, Kusatsu, Japan) following the manufacturer’s instructions Thereafter, cellular RNA was quantified using NanoDrop (spectrometer, Thermo Fisher, Waltham, MA, USA) (260/280 = 1.8), and the cDNA was synthesized using an iScript^TM^ cDNA synthesis kit (Bio-Rad, Hercules, CA, USA) followed by qRT-PCR using the SYBR Green PCR Master Mix (Bio-Rad). We employed a CFX96^TM^ Quantitative Real-Time system with a total reaction volume of 20 µL. The reactions were incubated using either a Bio-Rad qRT-PCR 96-well or 8-well tube at 95 °C for 10 min, followed by 40 cycles of 95 °C for 30 s, 55 °C for 30 s, and 72 °C for 30 s. All PCRs were performed in triplicate, and the cycle number at which the reaction crossed the threshold cycle (C_t_) was determined for each gene. The relative amount of each gene was measured relative to GAPDH RNA using the 2^ΔCt,^ equation. Enlisted primer sequences are provided in Supplementary Table S3.

### 2.3. Cell Cycle Analysis

To analyze the various phases of the cell cycle, KATO III and N87 cells were treated with HDACi and transfected with E2F5 siRNA and negative control siRNA. A total of 5 × 10^5^ cells were transferred into 6-well plates 48 h post-transfection, washed with warm PBS and digested using trypsin. The reaction of trypsin was neutralized with DMEM followed by centrifugation at 1000 rpm for 5 min. Next, the cells were treated with 70% ice-chilled ethanol and placed at −20 °C overnight. The next day, after fixation and washing with cold PBS twice, cell pellets were resuspended in 500 µL PBS containing 50 mg/mL propidium iodide, 0.1 mg/mL RNase A, and 0.05% Triton X-100, and incubated at 37 °C for 30 min in the dark. Cell cycle distributions were detected using FACSCalibur (Becton Dickinson, San Jose, CA, USA). For cell-cycle analysis, *n* = 10,000 cells.

### 2.4. Western Blot Analysis

Western blot analysis was used to investigate the expression of the target protein. In brief, extracted total lysate from the cells was washed thrice with PBS and then lysed in lysis buffer (50 mM Tris-base (pH 7.5), 0.5 M NaCl, 1 mM EDTA (pH 8)), 1 mM β-mercaptoethanol, 1% NP40, 1% glycerol, and protease inhibitor (Roach Molecular Biochemical, Mannheim, Germany). The concentration of total cellular proteins was determined by the Bradford method (Bio-Rad, Hercules, CA, USA). Thereafter, the protein samples were resolved on 10–15% sodium dodecyl sulfate–polyacrylamide gel electrophoresis and then transferred onto a polyvinylidene difluoride membrane (Millipore, Billerica, MA, USA). The membranes were blocked with 5% skimmed milk for 1 h at room temperature and incubated with specific primary antibodies (listed in Supplementary Table S4) followed by washing using washing buffer three times for 10 min each. Next, membranes were probed with the respective secondary antibody for 1 h followed by washing three times for 7 min each. Finally, the blots were probed with enhanced chemiluminescent reagent (Millipore), and the Western blot membranes were scanned using an iBright 1500 luminescent image analyzer (Thermo Fisher, Waltham, MA, USA).

### 2.5. Wound-Healing Assay

A wound-healing assay was executed to determine the migration rate of KATO III and N87 cells treated with HDACi and transfected with E2F5 siRNA and negative control siRNA. To accomplish this, cells were first digested, and then the concentration of cells was adjusted. The cell suspension was added to the cell culture plate for normal incubation until the formation of a cell monolayer. Then, the scratch assay was performed. Using a sterile 200 µL tip, three separate injuries through the cell monolayer were created per well, and the cells were then cultured in complete DMEM for next 24 h followed by determination of the comparative distance that the cell migrated to the injured area. Then, cell migration distance was determined by the following formula: cell migration = scratch distance at the beginning of experiment- scratch distance at the end of experiment. Injured area closure was monitored at intervals of 12 h, and images were captured using phase-contrast microphotography (200× magnification per field).

### 2.6. Cell Viability Assay

Cell viability curves are mostly observed using MTT (3-(4, 5-dimethylthiazol-2-yl)-2, 5-diphenyltetrazolium bromide) assay to determine the proliferation of treated cells. After 48 h transfection, cells were washed with warm PBS and trypsin was added to the cells. Then they were placed in incubator for 2–3 min to detach the cells from surface of culture dish. Then, fresh culture medium was added to neutralize the trypsin. The cells were transfected to a tube and centrifuged at 1000× *g* for 10 min. Then, the supernatant was discarded, and the pellet was dissolved in fresh medium. Cells were counted down with a hemocytometer in each group, and then inoculated into four 96-well plates at a density of 2 × 10^2^ cell per 200 µL DMEM with 8 repeated walls. After 24 h, the MTT solution was added to each well and incubated at 37 °C for 4 h. The reading was taken at intervals of 24 h. The incubation was stopped, and the culture supernatant was discarded. Dimethyl sulfoxide (DMSO) was added to each well, and the plates were shaken for 10 min in an enzyme-linked immunosorbent detector. The absorbance (OD) value of each well was measured at wavelength of 490 nm at each point. Cell viability curves were determined with time as the x-axis and OD values as the y-axis.

### 2.7. Statistical Analysis

Each of the experimental results was revised separately three times with each performed in triplicate. Statistical analysis of data was carried out using the GraphPad Prism 7 software (GraphPad Software, La Jolla, CA, USA). ANOVA and Student’s t test were used. All the numerical data were determined as the mean ± standard deviation, and *p* values < 0.05 were considered statistically significant for all comparisons. The difference was found to be statistically significant (* *p* < 0.05, ** *p* < 0.005, *** *p* < 0.001*)*.

## 3. Results

### 3.1. Investigation into Expression of HDACs in Gastric Cancer Cell Line and Normal Cell Line

**Figure 1:**
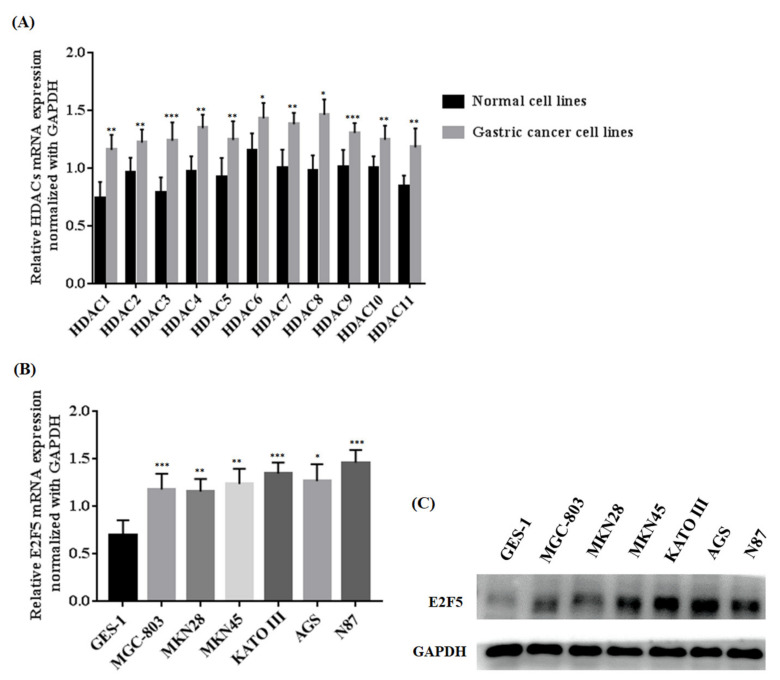
Determination of HDACs and E2F5 expression in normal (GES-1) and gastric cancer cell lines (MGC-803, MKN28, MKN45, KATO III, AGS, and N87). (**A**) Represents relative mRNA expression of HDACs (**B**) Represents relative mRNA expression of E2F5 (**C**) Western blot results show the protein expression of E2F5 in normal and gastric cancer cell lines using GAPDH as internal control. *: *p* < 0.05, **: *p* < 0.005, ***: *p* < 0.001.

Histone deacetylations play an important role in the regulation of different genes that are mainly related to the progression and development of human cancer. To explore the function of HDACs in gastric tumors, we investigated the expression of HDACs and E2F5 at both mRNA and protein levels in gastric cancer cell lines (MGC-803, MKN28, MKN45, KATO III, AGS, and N87) and a normal cell line (GES-1). The qRT-PCR results concluded that the relative mRNA expressions of HDACs in gastric cell lines were remarkably increased when compared with the normal cell line (* *p* < 0.05) (Figure 1A). Similarly, the present study also determined the mRNA expression of E2F5 in gastric cancer cell lines. The results revealed that relative mRNA expression of E2F5 was higher in gastric cancer cell lines compared with normal cell lines (* *p* < 0.05) (Figure 1B). For further confirmation, the Western blot results show that the protein expression of E2F5 was significantly higher in gastric cancer cell lines compared with the normal cell lines (Figure 1C). Such consequences suggest that HDAC and E2F5 might act as crucial biomarkers in gastric cancer.

### 3.2. Histone Deacetylase Inhibitor (HDACi) Downregulated the Expression of HDACs and E2F5 in Gastric Cell Lines

**Figure 2:**
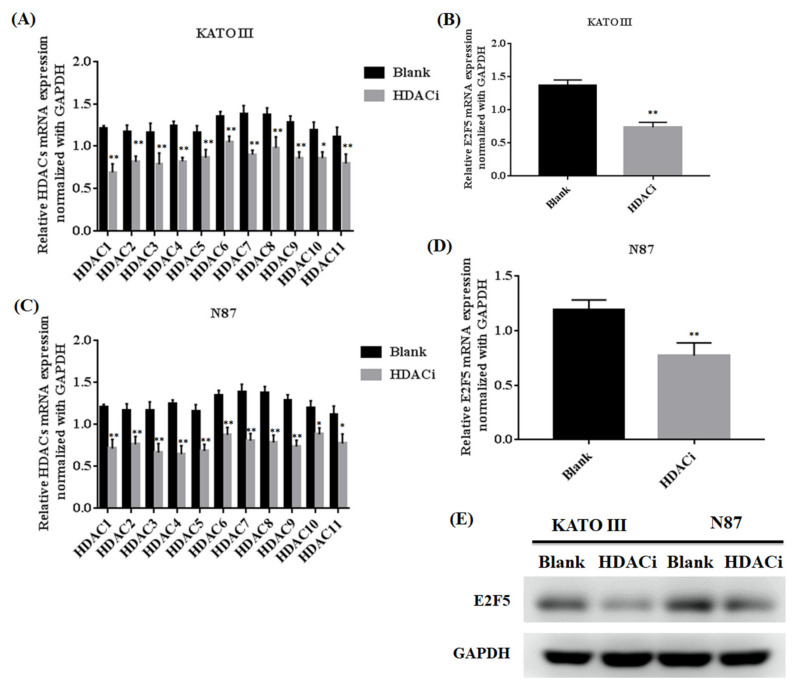
Histone deacetylase inhibitor (HDACi) downregulated the expression of HDACs and E2F5 in gastric cancer cell lines. (**A**) Represents relative mRNA expression of HDACs member in KATO III cell lines. (**B**) Represents relative mRNA expression of E2F5 in KATO III cell line. (**C**) Represents relative mRNA expression of HDACs member in N87 cell line. (**D**) Represents relative mRNA expression of E2F5 in N87 cell line. (**E**) Western blot result showed the protein expression of E2F5 in KATO and N87 III cell line using GAPDH as internal control. *: *p* < 0.05, **: *p* < 0.005.

Histone deacetylase inhibitor (HDACi) plays an important role in inhibiting the expression of HDACs in different human cancers. The gastric cell lines (KATO III and N87) were treated with HDACi to investigate the expression of HDACs and E2F5 at both mRNA and protein levels. The qRT-PCR results showed that HDACi downregulated the mRNA expression of HDACs in KATO III (** *p* < 0.005) and N87 (** *p* < 0.005) cell lines compared with the blank as a control (Figure 2A–D). Similarly, the results also showed that HDACi decreased the mRNA expression of E2F5 in KATO III (** *p* < 0.005) and N87 (** *p* < 0.005) cell lines compared with the blank (Figure 2A–D). For further confirmation, the Western blot results showed that HDACi decreased the protein expression of E2F5 in KATO III and N87 cell lines compared with the blank as a control (Figure 2E). Moreover, HDACs mRNA expression was significantly high in poorly differentiated N87 cell lines compared to other moderately differentiated KATO-III cell lines. The results revealed that HDACs regulate the expression of E2F5 gastric cancer cell lines.

### 3.3. HDACi Inhibited Cell Proliferation, Cell Migration, and Blocks Cell Cycle at G1 Phase via Downregulating E2F5 in Gastric Cell Lines

**Figure 3:**
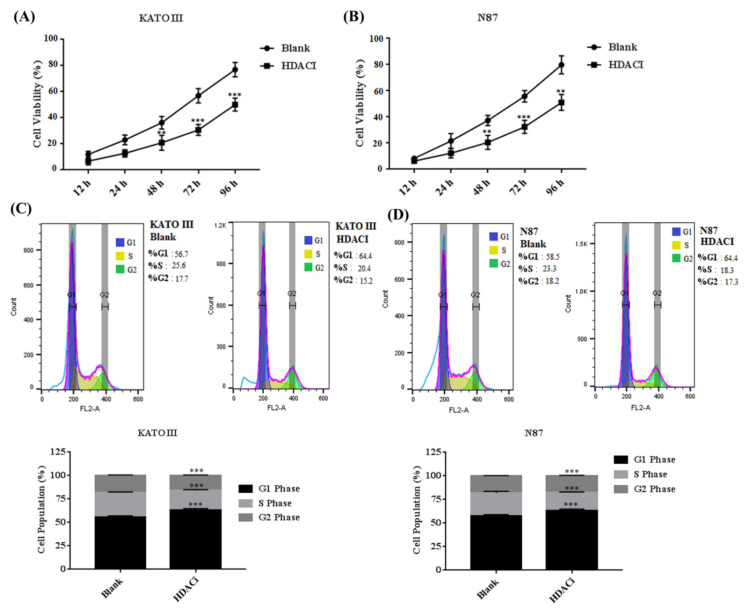
Histone deacetylase inhibitor (HDACi) decreased the cell proliferation in gastric cancer cell lines. (**A**) Represents the cell viability in KATO III cell line, (**B**) Represents cell viability in N87 cell line, (**C**) Represents cell cycle in KATO III cell line, (**D**) Represents cell cycle in N87 cell line compared to blank as a control. **: *p* < 0.005, ***: *p* < 0.001.

**Figure 4:**
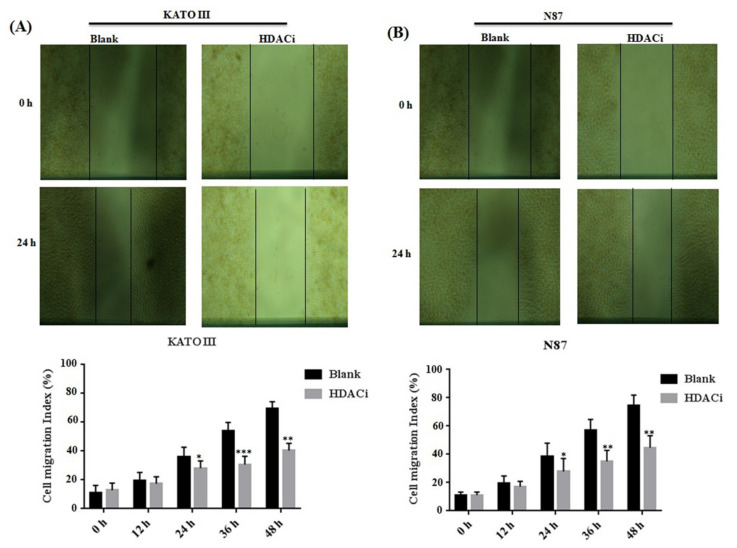
Histone deacetylase inhibitor (HDACi) decreased the cell migration in gastric cancer cell lines. (**A**) Represents the cell migration in KATO III cell line and (**B**) Represents cell migration in N87 cell line compared to blank as a control. *: *p* < 0.05, **: *p* < 0.005, ***: *p* < 0.001.

Aberrant expression of HDACs results in promoting the expression of E2F5, which mainly leads to increased cell proliferation and migration in different human cancers. The KATO III and N87 cell lines were treated with HDACi to determine the cell proliferation and cell cycle. The MTT results showed that HDACi inhibited cell proliferation in KATO III (** *p* < 0.005) and N87 (** *p* < 0.005) cell lines when compared with the blank as a control (Figure 3A,B). In addition, the flow cytometry results showed that HDACi decreased the number of the cells at the S phase via increasing the number of cells at the G1 phase of the cell cycle in KATO III (*** *p* < 0.001) and N87 (*** *p* < 0.001*)* cell lines, by downregulating the expression of E2F5 in comparison to the blank (Figure 3C,D). The results were statistically highly significant. Furthermore, a scratch assay was performed to determine cell migration in KATO II and N87 cell lines. The results showed that suppression of HDACs decreased cell migration in KATO III and N87 cell lines compared with the blank (* *p* < *0.05*) (Figure 4A,B).

### 3.4. Knocking Down of E2F5 Decreased the Expression of E2F5 and BCL2 in Gastric Cell Lines

**Figure 5:**
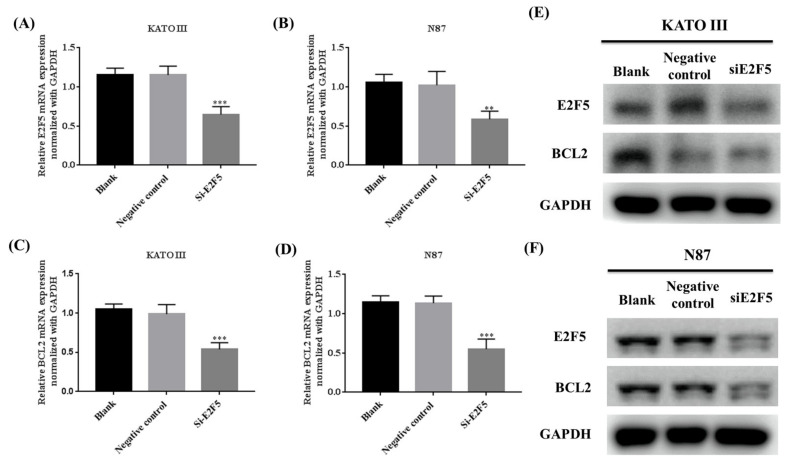
Knocking down of E2F5 downregulated the expression E2F5 and BCL2 in gastric cancer cell lines. (**A**) Represents relative mRNA expression of E2F5 in KATO III cell line. (**B**) Represents relative mRNA expression of E2F5 in N87 cell line. (**C**) Represents relative mRNA expression of BCL2 in KATO III cell line. (**D**) Represents relative mRNA expression of BCL2 in N87 cell line. (**E**) Western blot results showed the protein expression of E2F5 and BCL2 in KATO III cell line. (**F**) Western blot results showed the protein expression of E2F5 and BCL2 in N87 cell lines using GAPDH as internal control. **: *p* < 0.005, ***: *p* < 0.001.

E2F5 plays a significant role in the progression and development of human cancer. Alteration in the expression of E2F5 results in the activation of the expression of BCL2 in gastric cancer cell lines. The KATO III and N87 cell lines were transfected with E2F5 target siRNA and negative control (NC) siRNA to determine the expression of E2F5 and BCL2 at both RNA and protein levels. The qRT-PCR results revealed that knocking down of E2F5 decreased the mRNA expression of E2F5 and BCL2 in KATO III cell line (*** *p* < 0.001, *** *p* < 0.001) (Figure 5A,C) and N87 cell line (** *p* < 0.005, *** *p* < 0.001) ((Figure 5B,D) compared to negative control. The results were highly significant. The Western blot results showed that knocking down of E2F5 decreased the protein expression of E2F5 and BCL2 in gastric cancer cell lines compared with the negative control (Figure 5E,F).

### 3.5. Knocking Down of E2F5 Decreased Cell Proliferation, Migration, and Block Cell Cycle at G1 Phase in Gastric Cell Lines

**Figure 6:**
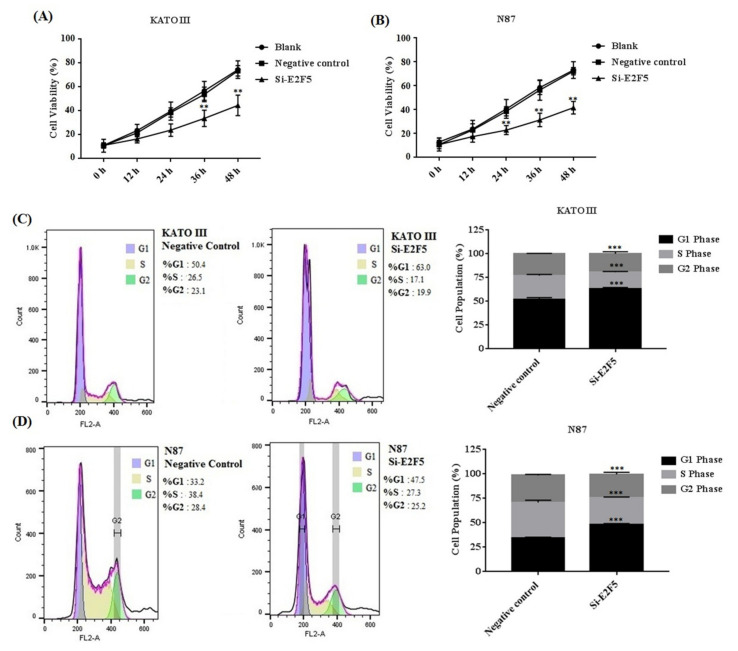
E2F5 knockdown decreased the cell proliferation in gastric cancer cell lines. (**A**) Represents the cell viability in KATO III cell line. (**B**) Represents cell viability in N87 cell line. (**C**) Represents cell cycle in KATO III cell line. (**D**) Represents cell cycle in N87 cell line when compared to negative control. **: *p* < 0.005, ***: *p* < 0.001.

**Figure 7:**
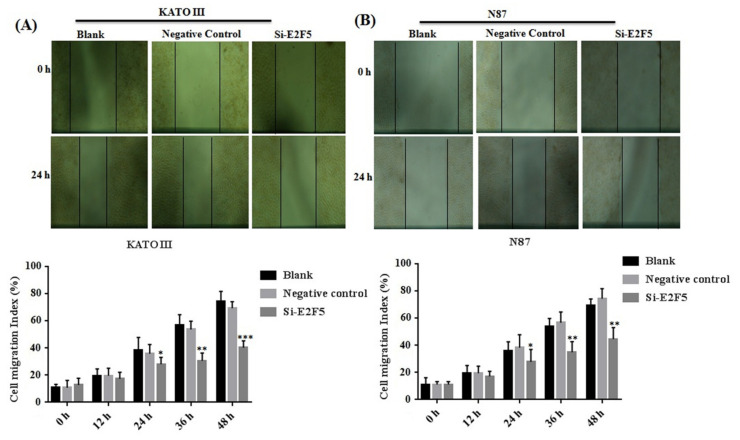
Knocking down of E2F5 decreased the cell migration in gastric cancer cell lines. (**A**) Represents the cell migration in KATO III cell line and (**B**) represents cell migration in N87 cell as compared to negative control. *: *p* < 0.05, **: *p* < 0.005, ***: *p* < 0.001.

E2F5 acts as an important transcription factor that mainly regulates cell proliferation and migration in different human cancers. Taken together, the results of the MTT assay revealed that knocking down of E2F5 decreased cell viability in KATO III (** *p* < 0.005) and N87 (** *p* < 0.005) cell lines compared with the negative control (Figure 6A,B). In addition, flow cytometry results showed that knocking down of E2F5 blocks the cell cycle at the G1 phase by increasing the number of the cell at the G1 phase via decreasing the number of the cell at S phase of cell cycle in KATO III and N87 cell lines, when compared with the negative control (* *p* < *0.05*) (Figure 6C,D). In addition, the results of scratch experiments determine that knocking down of E2F5 decreased cell migration in KATO III and N87 cell lines when compared to the negative control (* *p* < *0.05*) (Figure 7A,B).

## 4. Discussion

In the current study, we demonstrated the expression of HDAC through E2F5 targeting BCL2 downregulation, contributing a novel validation of the mechanism and treatment of HDAC inhibition. HDAC utilizes their targeted measures through post-translation acetylation of core nucleosome histones, accordingly, to modulate the expression of the target gene [12]. This anticancer regulatory mechanism of HDAC inhibitor in gastric tumors’ residue is imperfectly understood. Earlier investigations have explained that the suppression of distinct membranes of the HDACs family membrane acts as a latent suppressor, which is related to the growth, cell migration, proliferation, and invasion of gastric cells [13,14]. Interestingly, our results show that the expression levels of HDACs were remarkably increased in cell lines of gastric cancer. However, the involvement of HDAC inhibitors downregulated the expression levels of E2F5 at both the mRNA level and protein level. Let-7c downregulated the expression of E2F5 which blocks cell growth, invasion, proliferation, and migration in glioma cells [15]. Previous studies observed the potential of the HDAC inhibitor in acting as a stimulator of new factors for cancer treatment, such that it may respond to gene expression and retard the growth and endurance of cancer cells [16,17].

We recognize E2F5 as a straight target of BCL2 in gastric cells. A notable increase in E2F5 at the protein level guides the ectopic expression of BCL2. More essentially, current research demonstrates that the HDAC inhibitor could reverse PELP-mediated suppression of MIR-200 family members, suggesting an uninterrupted association between the HDAC inhibitor and family members of miR-200 in breast tumors [16,17]. This study illustrated that HDACi downregulates the expression of HDACs and E2F5 in gastric cancer cell lines. HDACi crucially hampered the cell migration, proliferation, as well as the invasion of cells [18]. Moreover, the inhibition of HDAC appearance can relatively invalidate the E2F5-mediated results in gastric tumor cells, and re-establish cell proliferation invasion and migration. Therefore, these results demonstrate that the expression of HDACi may perhaps be involved in cancer development and progression in gastric tumor carcinogenesis [2]. The current research intensifies the idea that the lower expression of E2F5 exhausts the inhibitory function of HDAC inhibitor in gastric cells, suggesting that the interaction of E2F5 with BCL2 inhibitors plays a critical part in the anticancer proceedings [19].

Moreover, HDAC decreases cell proliferation, invasion, and migration by suppressing the expression of E2F5 and BCL2. We showed that the HDAC inhibitor was inadequate to completely neutralize the inhibitory effect of proliferation, migration, and invasion of gastric cancer cell lines. One viable cause is that HDACs inhibition fails to completely block the increased level of E2F5 induced via BCL2. Regarding earlier studies on ductal carcinoma, it was described that the upregulation of CRKL protein takes part in integrating signs for invasion, proliferation, and migration of widely virulent breast carcinoma cell lines, and disclosed a remarkable connection between increased proliferative breast cancers and low outputs [20]. Our findings showed that HDAC inhibitors E2F5 and BCL2 are the therapeutic candidates for gastric carcinomas, which agrees with the hypothesis of HDAC being overexpressed in gastric cancer cell lines. HDAC inhibitors explain the high acetylation level of histone residues and therefore influence the expression of the gene. In recent studies, supporting our in vitro discovery, the therapeutic efficacy of the HDAC inhibitor on human gastric carcinoma samples using the histocellullar drug replication evaluation has been shown [21]. Nevertheless, it remains to be revealed whether specific molecular defined subgroups can anticipate response or resistance to HDAC inhibitors. In addition, the implementation of potentially functional biomarkers still has various restrictions. In light of the above studies, this context described the predominant findings that E2F5 may potentially conquer the profile capacity and metastasis ability in gastric cancer, suggesting a latent role of E2F5 in gastric cancer treatment [21]

Even though the previous observation of E2F5 was altered in various cancer cells, the mechanisms have not been completely explained [22]. For the investigation of the mechanistic role of E2F5 in gastric cancer, we observed that the level of E2F5 in gastric cancer cell lines using qRT-PCR was appreciably upregulated. These findings suggest that the appearance of other modulations at the translational level, possibly by the action moderated by mRNA, leads to gene silencing, resulting in translational repression. Therefore, the abnormal expression profile of HDACs may be involved in promoting the progression of gastric cancer. Cancer cells are mostly abnormally proliferated and differentiated. The role of HDACi in the proliferation, suppression, and migration of gastric cancer cell lines was also investigated, and it was shown that silencing of HDACs may decrease the proliferation and increase the apoptosis of gastric carcinogenesis. However, HDACs affect the migration and specifically participate in the proliferation of gastric tumor cells. It should be noted that the molecular factors of gastric cancer are a research priority, as we are now experts able to customize our study towards dissimilar genomic modifications for every molecular study, thus facilitating personalized medicine.

## 5. Conclusions

In conclusion, the knowledge gained from our study is that HDAC inhibitors impede gastric tumor cell migration, proliferation, and suppression of other cellular functions of tumors by regulating E2F5 through direct BCL2 targeting. This aspect of HDAC inhibitors’ regulation is anticipated not only to widen our understanding of basic cellular occurrence but also to configure advanced mechanisms of therapeutic agents required for the therapy of human diseases such as cancers. Our data somewhat show the conception of gastric cancer clinical therapy via HDAC inhibitors, and suggest that the HDAC–E2F5–BCL2 signaling axis might be a new diagnostic indicator in gastric cancer. However, further studies are required to better elucidate the molecular intricacies and the clinical interest in gastrointestinal practice.

## Data Availability

The data presented in this study are available upon request from the corresponding author.

## Supplementary Materials

The following are available online at https://www.mdpi.com/article/10.3390/life11121425/s1, Table S1: Composition of Histone deacetylation inhibitor (HDACi), Table S2: List of siRNAs used in this study, Table S3: List of primers used in this study, Table S4: List of antibodies used in this study.
