# Supplementary material for: Inhibition of HDACs Suppresses Cell Proliferation and Cell Migration of Gastric Cancer by Regulating E2F5 Targeting BCL2
Source: Life (Basel). 2021 Dec 17;11(12):1425. doi: 10.3390/life11121425 (PMC8705834; doi:10.3390/life11121425)
Supplement: Supplementary file 1 [file life-11-01425-s001.zip › life-1483367-supplementary.pdf]

Supplementary Table S1. Composition of Histone deacetylation inhibitor (HDACi).

Ingredient	M.W.	100× Conc.	Target
Trichostatin	302.37	40uM	Class I/II HDAC
EX-527	248.71	1mM	SIRT
Nicotinamide	122.12	400mM	ClassIII HDAC
Sodium Butyrate	110.09	200mM	Class I/II HDAC

Supplementary Table S2. List of siRNAs used in this study.

Gene Name	5′–3′	siRNA sequence
E2F5	Sense	GCACCUUCUGGUACACAAC
	Antisense	GUUGUGUACCAGAAGGUGC
NC	Sense	UUCUCCGAACGUGUCACGUUUGUGC
	Antisense	GCACAAACGUGACACGUUCGGAGAA

Supplementary Table S3. List of primers used in this study.

Gene Name	Direction	5′–3′ sequence
**HDAC1**	Sense	CTACTACGACGGGGATGTTGG
	Antisense	GAGTCATGCGGATTCGGTGAG
**HDAC2**	Sense	TCCGCATGACCCATAACTTGC
	Antisense	CCGCCAGTTGAGAGCTGAC
**HDAC3**	Sense	GCAAGGCTTCACCAAGAGTCT
	Antisense	AGATGCGCCTGTGTAACGC
**HDAC4**	Sense	GGCCCACCGGAATCTGAAC
	Antisense	GAACTCTGGTCAAGGGAACTG
**HDAC5**	Sense	TCTTGTCGAAGTCAAAGGAGC
	Antisense	GAGGGGAACTCTGGTCCAAAG
**HDAC6**	Sense	AAGAAGACCTAATCGTGGGACT
	Antisense	GCTGTGAACCAACATCAGCTC
**HDAC7**	Sense	GGCGGCCCTAGAAAGAACAG
	Antisense	CTTGGGCTTATAGCGCAGCTT
**HDAC8**	Sense	TCGCTGGTCCCGGTTTATATC
	Antisense	TACTGGCCCGTTTGGGGAT
**HDAC9**	Sense	AGTAGAGAGGCATCGCAGAGA
	Antisense	GGAGTGTCTTTCGTTGCTGAT
**HDAC10**	Sense	CCAACGGGTTCTGTGTGTTCA
	Antisense	CAGGAGAAGTAAAGGACGCTG
**HDAC11**	Sense	CACGCTCGCCATCAAGTTTC
	Antisense	GAAGTCTCGCTCATGCCCATT
**E2F5**	Sense	GGGCTGCTCACTACCAAGTTC
	Antisense	CCTACACCTTTCCACTGGATACT
**BCL2**	Sense	GGTGGGGTCATGTGTGTGG
	Antisense	CGGTTCAGGTACTCAGTCATCC
**GAPDH**	Sense	GGAGCGAGATCCCTCCAAAAT
	Antisense	GGCTGTTGTCATACTTCTCATGG

Supplementary Table S4. List of antibodies used in this study.

Antibody Used	Species	Company	Cat No
E2F5	Rabbit Polyclonal antibody	Invitrogen	PA5-85578
BCL2	Rabbit Polyclonal antibody	ABclonal	A0208
GAPDH	Rabbit Polyclonal antibody	Proteintech	10494-1-AP
HRP Goat Anti-Rabbit IgG	Goat secondary antibody	Proteintech	B900210
